# *Candida lusitaniae* Breakthrough Fungemia in an Immuno-Compromised Adolescent: Case Report and Review of the Literature

**DOI:** 10.3390/jof6040380

**Published:** 2020-12-21

**Authors:** Athanasia Apsemidou, Miriam Antonie Füller, Evgeny A. Idelevich, Oliver Kurzai, Athanasios Tragiannidis, Andreas H. Groll

**Affiliations:** 1 2nd Pediatric Department, Aristotle University of Thessaloniki, AHEPA Hospital, GR-54636 Thessaloniki, Greece; sissyaps@gmail.com (A.A.); atragian@auth.gr (A.T.); 2 Infectious Disease Research Program, Center for Bone Marrow Transplantation and Department of Pediatric Hematology and Oncology, University Children’s Hospital Münster, D-48149 Münster, Germany; MiriamAntonie.Fueller@ukmuenster.de; 3 Institute of Medical Microbiology, University Hospital Münster, D-48149 Münster, Germany; Evgeny.Idelevich@med.uni-greifswald.de; 4 Friedrich Loeffler Institute of Medical Microbiology, University Medicine Greifswald, D-17475 Greifswald, Germany; 5 National Reference Center for Invasive Mycoses, Leibniz Institute for Natural Product Research and Infection Biology—Hans-Knoell-Institute, D-07745 Jena, Germany; okurzai@hygiene.uni-wuerzburg.de; 6 Institute for Hygiene and Microbiology, Julius Maximilian University of Würzburg, D-97080 Würzburg, Germany

**Keywords:** *Candida lusitaniae*, candidemia, resistance, breakthrough, infection, transplantation

## Abstract

*Candida lusitaniae* is a rare cause of candidemia that is known for its unique capability to rapidly acquire resistance to amphotericin B. We report the case of an adolescent with grade IV graft-vs.-host disease after hematopoietic cell transplantation who developed catheter-associated *C. lusitaniae* candidemia while on therapeutic doses of liposomal amphotericin B. We review the epidemiology of *C. lusitaniae* bloodstream infections in adult and pediatric patients, the development of resistance, and its role in breakthrough candidemia. Appropriate species identification, in vitro susceptibility testing, and source control are pivotal to optimal management of *C. lusitaniae* candidemia. Initial antifungal therapy may consist of an echinocandin and be guided by in vitro susceptibility and clinical response.

## 1. Introduction

Invasive opportunistic fungal diseases caused by *Candida* spp. are important causes of morbidity and mortality in immunocompromised pediatric patients, including those with cancer and following hematopoietic cell transplantation (HCT). Whereas *Candida albicans* has long been the most common species isolated from blood cultures, there is a steady increase in fungal infections caused by non-albicans *Candida* species [1,2]. Among the non-albicans *Candida* species, *Candida lusitaniae* is an uncommon pathogen that accounts for approximately 1% of isolates in large datasets of adult and pediatric patients with candidemia and other forms of invasive candidiasis [1,3]. However, apart from its potential to be intrinsically resistant, this *Candida* species is of special interest as it is capable of rapidly acquiring resistance to amphotericin B and can cause breakthrough infections or treatment failure in immunocompromised patients [4,5,6,7]. This report describes the case of a severely immunocompromised 17-year-old adolescent allogeneic HCT recipient with late onset grade IV graft-vs.-host disease (GvHD) and catheter-associated *C. lusitaniae* candidemia occurring under long-term treatment with therapeutic doses of liposomal amphotericin B.

## 2. Case Report

The patient was a 17-year-old male adolescent with precursor T acute lymphoblastic leukemia (ALL) and persistent molecular disease who had received an allogeneic bone marrow transplant (HSCT) from a matched unrelated donor after standard conditioning with total body irradiation, high dose etoposide, and anti-thymocyte globuline in October 2015. Post-transplant immunosuppression consisted of three doses of methotrexate 10 mg/msqu on days 1, 3, and 6, and cyclosporine A with a target trough concentration of 120–140 ng/mL. Following discharge with a newly implanted Port-a-Cath (Smiths Medical, Dublin, OH, USA) central venous device on day +40 post-transplant, the further course in the outpatient setting was essentially unremarkable until the end of March 2016, when the patient developed late-onset grade IV graft versus host disease (GvHD) of the skin and the gastrointestinal tract during cyclosporine taper, necessitating in-patient management. 

Since adjustment of cyclosporine dosage and initiation of high-dose (2 mg/kg/day) methylprednisolone failed to induce a response, the patient subsequently received three courses of weekly rituximab (4 April–2 May) in combination with three courses of basiliximab and infliximab (13 April–14 May), while baseline immunosuppression with cyclosporine A (target trough concentration: 120–140 ng/mL) and methylprednisolone (1 mg/kg/day) was continued. In addition to parenteral nutrition, the patient received trimethoprim/sulfamethoxazole 160 mg twice daily (BID) on two days per week and valaciclovir 500 mg BID for prophylaxis, as well as meropenem 1000 mg three times daily (TID) plus linezolid 600 mg BID and liposomal amphotericin B 3 mg/kg/day as empirical therapy for intermittent fever of unknown origin. 

On the 3rd of May, while the patient was on empirical antibacterial and antifungal therapy and intermittently granulocytopenic (absolute neutrophil count (ANC) < 500 cells/uL) for more than four weeks, he developed a new fever episode with a maximum temperature of 38.7 °C. The ANC on that day was 690 cells/mL, the hemoglobin was 7.7 g/dL, the platelet count was 16.000/uL, and the c-reactive protein (CRP) was 0.5 mg/dL. Total serum bilirubin was 1.7 mg/dL, and aspartate aminotransferase (AST), alanine aminotransferase (ALT), and alkaline phosphatase were 58, 62, and 169 U/L, respectively. Serum creatinine, blood urea nitrogen (BUN), serum albumin, serum electrolytes, and blood sugar were within normal limits. Blood cultures taken during the initial fever episode remained sterile for bacteria but grew yeasts that were subsequently identified as *Candida lusitaniae* by MALDI-TOF MS and confirmed by sequencing of the ITS1-5.8S-ITS2 region [8]. Susceptibility testing was performed by broth microdilution according to the EUCAST reference method [9,10], revealing increased minimum inhibitory concentration (MIC) of amphotericin B and no evidence for reduced susceptibility towards azoles or echinocandins (Table 1), with the theoretical limitation that resistance testing of sessile *C. lusitaniae* cells in biofilms was not performed. Treatment with caspofungin at a dose of 50 mg/day (day 1: 70 mg) was initiated on 6 May and liposomal amphotericin B continued at 3 mg/kg/day to maintain coverage for filamentous fungi. 

**Figure 1:**
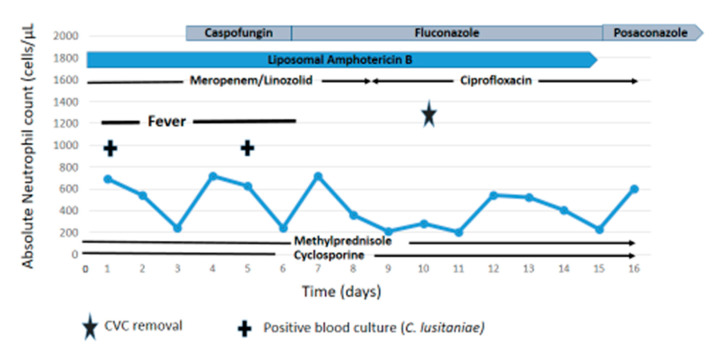
Time course of *Candida lusitaniae* fungemia and antifungal management.

While the patient remained hemodynamically stable, he had persistent intermitting fever episodes that were monitored by daily blood cultures. Whereas there was no growth on days 2 to 4, blood cultures obtained on day 5 became positive for *C. lusitaniae* with an in vitro susceptibility pattern similar to the initial isolate (Table 1). Blood counts, blood sugar, serum electrolytes, and parameters of renal and hepatic function were essentially unchanged, and the CRP was 0.9 mg/dL (maximum: 3.8 on day 3). At this point, on day 7 (9 May), caspofungin was discontinued and antifungal therapy switched to intravenous fluconazole (400 mg once daily (QD); day 1: 800 mg) plus liposomal amphotericin B 3 mg/kg/day, with the indwelling central venous catheter (CVC) finally being removed on day 10 (12 May) (Figure 1). Following defervescence and exclusion of intraocular and intraabdominal lesions, antifungal treatment with fluconazole and liposomal amphotericin B was continued for 10 days after the last positive blood culture (17 May) and then replaced by oral posaconazole (300 mg QD; day 1: 300 mg BID of the gastroresistant tablets) as antifungal prophylaxis (Figure 1). Sadly, 1 month after completion of antifungal therapy and while in partial remission of active GvHD, the patient succumbed to a catheter-related Gram-negative bacterial bloodstream infection with sepsis and refractory septic shock without apparent causal relationship to the prior episode of *C. lusitaniae* fungemia and its treatment.

## 3. Discussion

While usually appearing susceptible to amphotericin B, the triazoles, and the echinocandins in vitro [1,13,14,15], *C. lusitaniae* is different from most medically important *Candida* species as it can readily develop in vivo resistance to amphotericin B upon exposure to this agent. Apart from mutations in the ergosterol biosynthetic pathway that may have direct effects on gene expression in this haploid yeast, selective gene expression in the adaptive response to amphotericin B may occur with high frequency reversible phenotypic switching from susceptibility to resistance associated with distinct morphologies [5,16,17,18]. Rapid switching to a resistant phenotype with markedly reduced fungicidal activity has been induced in vitro in susceptible strains from patients with failure of amphotericin B-based treatment, which may contribute to the lack of consistent correlations between in vitro susceptibility and responses in vivo [5]. Development of cross resistance to fluconazole in the switched phenotype [19] and of multidrug resistance with accumulation of mutations conferring resistance to all antifungal agents [6,7] have been observed in individual patients. These observations illustrate the potential of *C. lusitaniae* to rapidly adapt to drug pressure within the host and emphasize the particular need for careful monitoring of antifungal susceptibility and treatment responses.

Dedicated clinical data on invasive *C. lusitaniae* infections are scarce. In a literature review published in 2003, a total of 55 cases were reported with a predominance of bloodstream infections (80%). Although three-fourths of the patients had serious underlying medical conditions, attributable mortality was low (5%). A total of 5 (21.7%) of 23 isolates with susceptibility testing data were resistant to amphotericin B; two of the affected patients were cured with amphotericin B treatment, and all five survived [20]. In a retrospective analysis conducted at M. D. Anderson Cancer Center in Houston, Texas, between 1988 and 1999, 12 cases of *C. lusitaniae* candidemia were identified, of which 7 occurred as breakthrough infection (58%; four on amphotericin B and three on fluconazole). Eight patients had hematologic malignancies or were post-HCT, and most patients (75%) were granulocytopenic at the time of infection. Amphotericin B alone failed in three of six patients; fluconazole alone was effective in three patients with solid tumors, and amphotericin B plus fluconazole was effective in two of three patients. None of the nine isolates available from five patients were resistant in vitro (MIC > 1 mg/L) to amphotericin B. The case fatality rate was 25% [21]. In a subsequent study from the same institution covering 1998 through 2013, *C. lusitaniae* was isolated in 19 of 1395 *Candida* bloodstream isolates (1.4%; or 28% among 68 patients with fungemia by uncommon *Candida* species, including *Candida guilliermondii, Candida lusitaniae*, *Candida kefyr*, *Candida famata*, and *Candida dubliniensis*), with a significant increase over time. Most patients had hematologic malignancies (75%), 18 were post-HCT (27%), and 40 (58.8%) were severely granulocytopenic; a total of 7 of the 19 episodes (36.8%) occurred as breakthrough infections. All 19 isolates had an MIC of amphotericin B of ≤1 mg/L, and 3 of 14 tested isolates had an MIC of caspofungin of >1 mg/L as assessed by the Clinical Laboratory Standards Institute broth microdilution reference method [22]. The all-cause 28-day mortality rate was 53% and not different from the entire cohort [23].

Two large epidemiological studies have been conducted in pediatric patients. In a prospective, multicenter observational study of invasive candidiasis conducted by the International Pediatric Fungal Network between 2007 and 2011, *C. lusitaniae* accounted for 8 of 201 isolates (4%) collected from 196 non-neonatal pediatric patients; two of the eight cases were breakthrough infections [24]. In a similar multinational but retrospective study of candidemia conducted by the European Pediatric Mycology Network covering 2005 through 2015, *C. lusitaniae* was found in less than 2% of the 1395 cases of candidemia collected in pediatric patients of all age groups [25]. Data obtained from our own institution, the University Children’s Hospital Münster, between 1998 and 2016, revealed 56 episodes of candidemia in patients below 20 years. The majority were immunocompromised or critically ill (75%), had an indwelling central venous catheter (92%), and were receiving broad spectrum antibacterial agents (94%) at diagnosis. Non-*albicans Candida* species accounted for 52% of the isolates, with *Candida parapsilosis* accounting for 19.6%, *Candida glabrata* for 10.7%, and *C. lusitaniae* for 7.1%. Of the four primary isolates of *C. lusitaniae*, two were resistant to amphotericin, two to fluconazole, and one was resistant to 5-fluorocytosine; one of the four cases (the presented case) was a breakthrough infection under amphotericin B therapy [26,27].

Breakthrough fungal infections have been recognized as an emerging problem [28,29] and are defined as any invasive fungal infection occurring during exposure to a systemic antifungal agent, irrespective of its spectrum of activity [30]. In a retrospective study conducted between 2011 and February 2016 in patients >12 years admitted to two tertiary care hospitals in Southern Brazil, 27 breakthrough episodes (18%) were identified among 148 candidemia episodes. Breakthrough infections were associated with neutropenia and mucositis by multivariate analysis and showed a predominance of *Candida* non-*albicans* species (85%; *p* < 0.001). There was no clear pattern of resistance in the breakthrough isolates, and no difference was observed in 30 days mortality; *C. lusitaniae* was not observed among the breakthrough infections [29]. In a retrospective single center study conducted in a pediatric tertiary care center in Taiwan from 2003 through 2015, 45 of 319 episodes of candidemia (14.1%) were breakthrough infections. Similar to the previous study, the majority of breakthrough candidemia was caused by non-*albicans Candida* species (73.3%; *p* < 0.01), but only 5 of 43 isolates studied (11.6%) were resistant to the antifungal agent used at the time of breakthrough. Isolation of *C. lusitaniae* was sporadic (<4%), without apparent differences in breakthrough relative to non-breakthrough candidemia. By multivariate analysis, previous azole exposure, neutropenia, and recurrent candidemia were independent risk factors of breakthrough candidemia; episodes of breakthrough candidemia had significantly higher illness severity (*p* < 0.01) and higher rates of attributable mortality (*p* < 0.001) [31].

The patient presented here had classical risk factors for breakthrough candidemia, including hematologic malignancy, status post-allogeneic matched unrelated donor (MUD) HCT, and severe GvHD, prolonged granulocytopenia, impaired mucosal barriers, profound immunosuppression including glucocorticosteroids, presence of a central venous catheter, and long-term use of broad-spectrum antibacterial agents [32]. Similar to the findings in the two larger studies described above [29,31], the isolate belonged to the group of non-*albicans Candida* species and, in this specific case, was phenotypically resistant to amphotericin B, which had been used as empirical antifungal treatment over prolonged periods of time. However, given the clinical predominance of predisposing host and iatrogenic factors, the finding of antifungal resistance and its role in the pathogenesis of this infection are difficult to assess [32]. Clinical non-response to caspofungin, as suggested by persistent fever and two separate positive follow-up blood cultures on day 5 of treatment, most likely was not attributable to failure of the echinocandin but to the lack of source control, as evidenced by prompt improvement and clinical cure following removal of the indwelling central venous catheter. Of note, in an individual patient-level quantitative review of seven randomized trials for treatment of invasive candidiasis including data from 1915 adult patients, removal of a central venous catheter (*p* = 0.0001) and treatment with and echinocandin antifungal (*p* = 0.02) were associated with clinical success and survival [3], leading international guidelines to strongly recommend first-line treatment with an echinocandin and catheter removal for pediatric patients also [33,34,35].

In summary, the haploid yeast *C. lusitaniae* is a rare cause of invasive candidiasis in immunocompromised children and adolescents that may readily develop resistance to amphotericin B and, potentially, the triazoles and the echinocandins. Careful attention to appropriate species identification, discriminative in vitro susceptibility testing, and strict adherence to the principle of source control are pivotal to optimal management. In the absence of more robust clinical data, initial antifungal therapy should probably consist of an echinocandin and thereafter be guided by the results of in vitro susceptibility testing and clinical response.

## Figures and Tables

**Table 1:** In vitro susceptibility of the patient’s isolates.

Antifungal Agent	Minimum Inhibitory Concentration (mg/L)		
	Day 1 Isolate JMRC:NRZ:0688	Day 5 Isolate JMRC:NRZ:0689	Day 5 Isolate JMRC:NRZ:0690
Amphotericin B	1	1	2
Fluconazole	≤0.125	≤0.125	≤0.125
Itraconazole	0.125	0.125	0.125
Voriconazole	≤0.016	≤0.016	≤0.016
Posaconazole	≤0.016	≤0.016	≤0.016
Anidulafungin	0.06	0.03	0.03
Caspofungin	0.5	0.5	1

For details on clinical breakpoints, please see references [10,11,12]. Day 5 isolates stemmed from different blood culture bottles; the MIC of amphotericin B of isolate 0690 was above the epidemiological cut off value (ECV) of 1 mg/L [11], and the MIC of caspofungin was above the ECV of 0.5 mg/L [12], indicating reduced susceptibility to both agents.
